# Migraine Prevalence and Academic Impact on Medical Students at Alfaisal University

**DOI:** 10.1002/brb3.70072

**Published:** 2024-10-23

**Authors:** Anikó Szabó, Golam Mahamud, Faridul Ahsan

**Affiliations:** ^1^ Department of Anatomy and Genetics, College of Medicine Alfaisal University Riyadh Saudi Arabia; ^2^ College of Medicine Alfaisal University Riyadh Saudi Arabia

**Keywords:** academic performance, coping strategies, medical students, migraine, prevalence

## Abstract

**Background:**

Migraine is highly prevalent among medical students but has been understudied in Saudi Arabia. This study aimed to determine migraine prevalence, characteristics, academic impacts, and coping strategies in medical students.

**Methods:**

A cross‐sectional survey was conducted among 352 medical students. Migraine was identified using ID‐Migraine (38.8% prevalence) and International Classification of Headache Disorders 3rd edition (ICHD‐3) criteria (36.8% prevalence). Analyses focused on the 130 students meeting ICHD‐3 criteria.

**Results:**

The mean monthly migraine frequency was 3.5 days. Average pain severity was moderate at 6.4/10. Mean duration was 9.3 h. Exams triggered migraines for 66% and increased headache frequency/severity for most students. Headaches limited productivity ≥1 day for 86% and caused missed exams and grade point average (GPA) declines. Rest (77.7%), trigger avoidance (57.7%), and medications (56.9%) were the most common coping strategies.

**Conclusion:**

Migraine prevalence exceeded general population estimates, frequently disrupted academics, and was exacerbated by exam stressors. Support services and education on coping strategies tailored to academically triggered migraines could better equip students to manage headaches.

## Graphical abstract

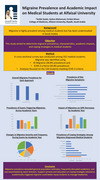

## 1 Introduction

Migraine is a chronic neurological disorder characterized by recurrent moderate‐to‐severe headaches, often unilateral and pulsating in nature (Headache Classification Committee of the International Headache Society (IHS) 2018). It affects approximately 11% of the global population and is one of the leading causes of disability worldwide (2016 Headache Collaborators 2018). Migraine has a particularly high prevalence among university students, with rates ranging from 10% to 25% in this population (Smitherman et al. 2013; Woldeamanuel and Cowan 2017). The high cognitive demands and academic stressors experienced by university students are believed to act as triggers for migraine attacks (Waldie et al. 2002). This susceptible population frequently reports decreased academic performance, increased school absences, and disruption of daily activities due to migraines (Bigal et al. 2001; Smitherman et al. 2013; Souza‐e‐Silva and Rocha‐Filho 2011). Medical students, particularly those in their final years of study, may be at higher risk for more frequent and severe migraines, possibly due to increased stress levels, irregular sleep schedules, and demanding coursework (Al‐Hashel et al. 2014). Previous studies have found migraine prevalence rates of 28.3% among medical students in India (Menon and Kinnera 2013), 27.9% in Kuwait (Al‐Hashel et al. 2014), and 28.5% in Brazil (Ferri‐de‐Barros, Alencar, and Berchielli 2011). However, the epidemiology of migraine in medical students in Saudi Arabia has not been extensively investigated. A study by Ibrahim et al. (2017) reported a migraine prevalence of 26.3% among medical students at King Abdulaziz University in Jeddah, Saudi Arabia. Given the limited data on migraine among medical students in Saudi Arabia, this study aims to determine the prevalence, characteristics, and academic impacts of migraine in medical students at Alfaisal University. We will also analyze associations between academic stressors like examinations and migraine patterns. Findings will provide insights into migraine burden and triggers among this understudied Saudi medical student population. The data can inform institutional efforts to support migraine management during academically intensive periods for this high‐risk cohort.

## 2 Methods

### 2.1 Participants and Study Design

A cross‐sectional, questionnaire‐based study was conducted among medical students at Alfaisal University. The study population included all actively enrolled medical students from Years 1 to 6 during the 2023–2024 academic year. The survey was distributed electronically to approximately 1000 medical students using the university's student email system. The survey was hosted on Google Forms, which allowed for anonymous data collection and enabled branching logic.

### 2.2 Inclusion and Exclusion Criteria

All actively enrolled medical students from Years 1 to 6 at Alfaisal University during the 2023–2024 academic year were eligible to participate in the study. There were no specific exclusion criteria applied. However, participants with missing data were excluded from the analysis.

This study employed a novel approach to migraine identification by combining the ID Migraine screening tool and the International Classification of Headache Disorders 3rd edition (ICHD‐3) criteria. The use of both methods enhances the accuracy of the migraine prevalence estimate compared to studies using only one method.

### 2.3 Migraine Identification

A stepwise approach was used to identify migraine cases. Students were first asked, “In the past 3 months, have you had headaches lasting 4 to 72 hours?” Those answering affirmatively were evaluated for probable migraine based on ID Migraine (Lipton et al. 2003) and ICHD‐3 criteria (Headache Classification Committee of the International Headache Society (IHS) 2018). ID Migraine criteria were assessed through the question, “During these headaches, did you experience any of the following features? (check all that apply): Nausea and/or vomiting, Bothering you (Increased sensitivity to light and sound), Limited your ability to work/study for at least one day.” Students endorsing two or more items have a sensitivity of 81% and specificity of 75% for identifying migraines in primary care settings based on the ID Migraine criteria (Lipton et al. 2003). ICHD‐3 criteria were examined through the following questions: “In the past 3 months, have you had headaches lasting 4–72 hours that were not relieved by over‐the‐counter medication?,” “Does it have more than 5 attacks? Yes or No,” and “Did these headaches have at least 2 of the following features? (check all that apply): Throbbing, pulsating pain, Pain on one side of the head, Moderate to severe intensity, Worsened by routine activity like walking.” Students who met all the criteria up to this point were evaluated with the question “Have you ever received a medical diagnosis of migraine headaches by a doctor?” to rule out other potential causes of headaches. Students meeting ICHD‐3 were also classified as having probable migraine. To comprehensively capture all potential migraine cases, migraine prevalence was assessed using both ID Migraine (Lipton et al. 2003) and ICHD‐3 criteria (Headache Classification Committee of the International Headache Society (IHS) 2018). Students meeting both criteria were categorized as having a definite migraine. However, for all subsequent analyses, only students fulfilling ICHD‐3 criteria (Headache Classification Committee of the International Headache Society (IHS) 2018) considered for migraine diagnosis, were included. This focused the investigation specifically on students with migraines meeting established international criteria. Restricting these analyses to only ICHD‐3 migraine enabled the characterization of impacts and patterns uniquely related to clinically confirmed migraine based on the well‐established diagnostic criteria (Headache Classification Committee of the International Headache Society (IHS) 2018). Migraine severity was classified based on headache frequency using established guidelines (Headache Classification Committee of the International Headache Society (IHS) 2018). Headache intensity was measured on a 0–10 visual analog scale as recommended by the International Headache Society (Tfelt‐Hansen et al. 2012). Migraine frequency, severity, and duration were assessed with the following survey questions: “In the past 3 months, how many DAYS did you have a headache?,” “What is the average duration of your migraine headaches?” and “In the past 3 months, what was the average SEVERITY of your headache pain on a scale of no pain (zero) to worst pain (10)?” This methodology enabled systematic classification of migraine severity while adhering to International Headache Society standards for measurement of pain intensity and headache characteristics.

### 2.4 Academic Impacts

The effects of migraines on academic performance were evaluated in multiple ways. First, students were asked, “Do exams trigger your migraine headaches?” with a yes/no response to determine if exams acted as a trigger. Specific migraine symptoms and characteristics during exams were then assessed categorically for students meeting ICHD‐3 migraine criteria. Questions included: 1. “DURING exams, did you experience an increase in migraine headache frequency compared to other times?” (Yes/No) 2. “DURING exams, did you experience an increase in migraine headache intensity/severity compared to other times?” (Yes/No) 3. “What is the average duration of your migraine headaches DURING exams?” (<4, 4–12, 12–24, 24+ h). Finally, the impacts of migraine symptoms on academics were examined with questions on exam absences, lost productivity, and GPA changes: (1) “DURING exams, how many EXAMS did you miss due to migraine headaches?” (2) “DURING exams, how many DAYS did migraine headaches SIGNIFICANTLY LIMIT your productivity/concentration?” (3) “Did your GPA decrease from migraine headaches?” (Yes/No). Chi‐squared tests compared academic impacts across academic years. This multifaceted approach evaluated migraine triggers, symptom exacerbation, and disruption of academic performance during exam periods.

### 2.5 Statistical Analysis

The collected questionnaire data were analyzed using Statistical Package for the Social Sciences (SPSS) for Windows version 27 to assess migraine prevalence, clinical migraine characteristics, academic impacts, and coping strategies. Descriptive statistics summarized the sample. Chi‐squared tests compared categorical migraine variables like symptoms and academic impacts across demographic groups. One‐way ANOVA tested differences in numerical migraine characteristics like frequency, severity, and duration across gender, age, and academic year. Associations between academic impacts and demographics were evaluated using chi‐squared tests. *p* values below 0.05 were considered statistically significant. SPSS enabled thorough analysis of associations and group differences.

## 3 Results

### 3.1 Study Population

Out of 1000 students who received the invitation, 352 provided consent and completed surveys after excluding 13 with missing data, representing a 35.2% response rate. Across the questionnaire items, missing data were minimal, affecting less than 4% of the total sample, so we excluded them all. Table 1 presents a complete breakdown of the study's demographic composition, detailing both sample sizes and corresponding percentages. The majority were female (68.5%) and in early academic years, with first years most represented (44.5%). Age skewed young, with most participants 18–20 years old (61.1%). There were significant differences across gender, age groups, and academic years (all *p* < 0.001), indicating disproportionate representation. The sample reflects a convenience approach rather than probability sampling.

**TABLE 1:** Demographic characteristics of the study sample.

Demographic		Frequency	Percentage
Biological sex	Female	241	68.47
	Male	111	31.53
Age	Age 18–20	215	61.08
	Age 21–23	107	30.40
	Age 24–26	20	5.68
	Age 27+	10	2.84
Academic year	1st	157	44.60
	2nd	68	19.32
	3rd	55	15.62
	4th	29	8.24
	5th	23	6.53
	6th	20	5.68

### 3.2 Migraine Prevalence

**FIGURE 1:**
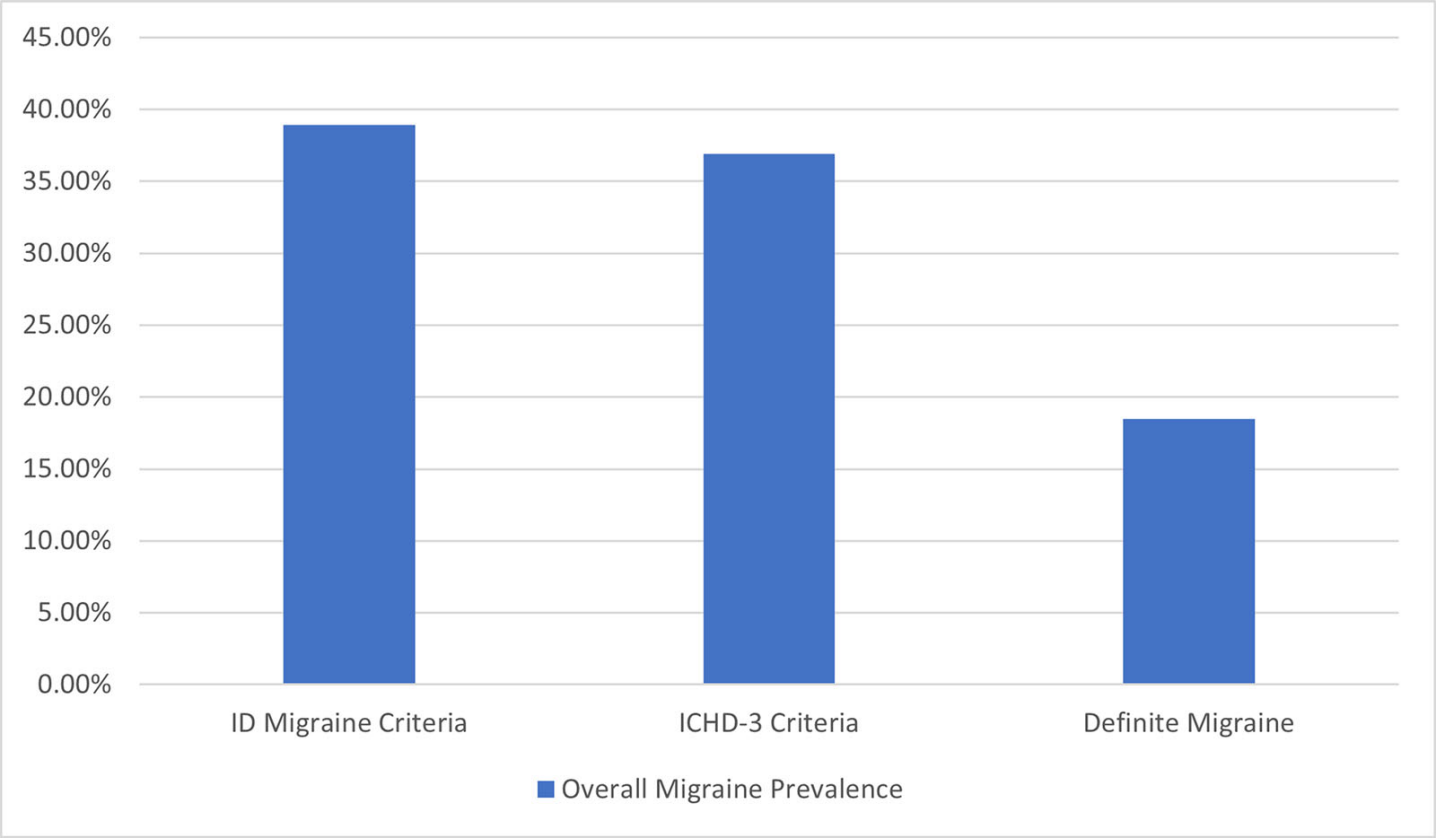
Overall migraine prevalence for each approach. ICHD‐3, International Classification of Headache Disorders 3rd edition.

The analysis identified three estimates of migraine prevalence based on different criteria. ICHD‐3 diagnosis, ID Migraine screening, and a definite migraine subgroup meeting both criteria. Figure 1 shows the overall prevalence percentages for each approach. Using the ICHD‐3 criteria, the migraine prevalence was 36.8%. The ID Migraine screening tool identified a prevalence of 38.8%. Further analysis identified a definite migraine prevalence subgroup meeting both ICHD‐3 and ID Migraine criteria, which provides a more conservative estimate requiring agreement across measures. This subgroup had a prevalence of 18.5%. There were no statistically significant differences in definite migraine prevalence based on gender (19.5% female vs. 16.2% male, *p* = 0.555), age (ranging from 17.7% to 20%, *p* = 0.972), or academic year (ranging from 12.7% to 25.0%, *p* = 0.638). Full details on the three migraine prevalence estimates and demographic comparisons are presented in Table 2.

**TABLE 2:** Prevalence of migraine by diagnostic criteria and demographics.

Variables		Definite migraine group				ID Migraine criteria				ICHD‐3 criteria			
		Prevalence (%)	Sample	*p* value	*χ* ^2^	Prevalence (%)	Sample	*p* value	*χ* ^2^	Prevalence (%)	Sample	*p* value	*χ* ^2^
Overall prevalence		18.47	65/352	N/A	N/A	38.92	137/352	N/A	N/A	36.93	130/352	N/A	N/A
Age	18–20	17.67	38/215	0.9723	0.2320	39.53	85/215	0.0576	7.497	36.28	78/215	0.1694	5.033
	21–23	19.63	21/107			32.71	35/107			39.25	42/107		
	24–26	20.00	4/20			65.00	13/20			20.00	4/20		
	27+	20.00	2/10			40.00	4/10			60.00	6/10		
Biological sex	Male	16.22	18/111	0.5549	0.3486	29.73	33/111	0.0225	5.210	31.53	35/111	0.1916	1.705
	Female	19.50	47/241			43.15	104/241			39.42	95/241		
Academic year	1st	18.47	29/157	0.6376	3.4062	40.13	63/157	0.8060	2.302	37.58	59/157	0.1219	8.693
	2nd	23.53	16/68			36.76	25/68			47.06	32/68		
	3rd	12.73	7/55			41.82	23/55			27.27	15/55		
	4th	17.24	5/29			27.59	8/29			44.83	13/29		
	5th	13.04	3/23			39.13	9/23			21.74	5/23		
	6th	25.00	5/20			45.00	9/20			30.00	6/20		

Abbreviation: ICHD‐3, International Classification of Headache Disorders 3rd edition.

### 3.3 Migraine Characteristics

**FIGURE 2:**
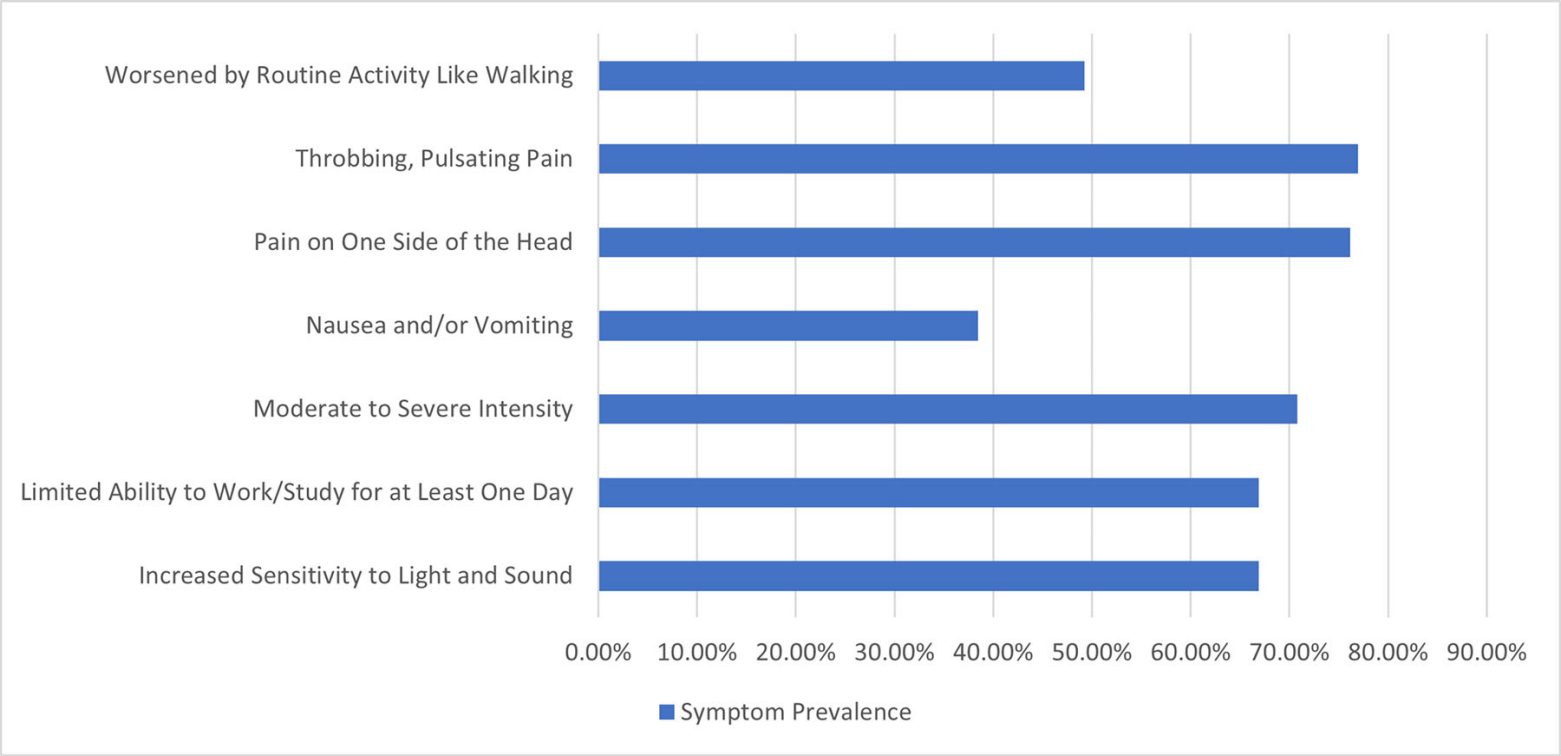
The prevalence of key migraine symptoms.

Among the 130 students meeting ICHD‐3 migraine criteria, frequency analysis found a mean of 3.5 migraine days per month (SD 1.6), with no significant differences by gender (*p* = 0.055), age (*p* = 0.118), or academic year (*p* = 0.476) (Table 3). For migraine severity, the mean rating was 6.4 (SD 2.1) on a 10‐point scale. No significant differences were detected by gender (*p* = 0.093), age (*p* = 0.061), or academic year (*p* = 0.825). The mean migraine duration was 9.3 h (SD 7.6), with significant differences across age groups (*p* = 0.029) but not gender (*p* = 0.892) or academic year (*p* = 0.372). Chi‐square analysis evaluated associations between specific migraine symptoms and demographic factors among the 130 respondents (Table 3). The symptom of moderate‐to‐severe headache intensity showed a significant association with the academic year (*χ* ^2^ = 17.84, *p* = 0.0032). No other symptoms demonstrated significant associations. Nausea and sensitivity to light/sound were common, reported by 67% and 76%, respectively. The throbbing pain was experienced by 77%. Prevalence was similar across groups. The symptom “limited ability to work/study” was not significantly associated with gender (*χ* ^2^ = 0.0010, *p* = 0.9742), age (*χ* ^2^ = 3.20, *p* = 0.3613), or academic year (*χ* ^2^ = 3.85, *p* = 0.5712). Figure 2 shows the prevalence of key migraine symptoms. Full details of migraine symptoms in the study population are presented in Appendix A1.

**TABLE 3:** Clinical migraine characteristics.

Variables		Overall	Biological sex		Age group				Academic year					
			Male	Female	18–20	21–23	24–26	27+	1st	2nd	3rd	4th	5th	6th
Distribution	*N* (%)	130 (100)	35 (26.9)	95 (73.1)	78 (60.00)	42 (32.30)	4 (3.1)	6 (4.6)	59 (45.4)	32 (24.6)	15 (11.5)	13 (10.0)	5 (3.9)	6 (4.6)
Frequency (days/month)	M ± SD	3.56 ± 1.54	3.03 ± 1.67	3.76 ± 1.45	3.45 ± 1.53	3.69 ± 1.55	2.25 ± 1.50	3.36 ± 1.62	4.13 ± 1.13	3.73 ± 1.62	3.46 ± 1.98	3.40 ± 0.89	2.50 ± 1.22	3.36 ± 1.62
	Range	0–5	0–5	0–5	0–5	0–5	0–5	5–5	0–5	1–5	0–5	0–5	3–5	0–3
	ANOVA *p* value	N/A	0.016		0.029				0.129					
Severity (Scales 0–10)	M ± SD	6.41 ± 2.22	5.80 ± 2.90	6.63 ± 1.87	6.13 ± 2.15	6.83 ± 2.25	5.00 ± 3.37	8.00 ± 0.00	6.34 ± 2.45	6.59 ± 1.46	6.27 ± 1.75	6.08 ± 2.96	7.80 ± 1.79	6.00 ± 3.03
	Range	0–10	0–9	0–10	0–10	0–10	0–7	8–8	0–10	3–10	4–9	0–9	5–9	0–8
	ANOVA *p* value	N/A	0.057		0.056				0.736					
Duration (h)	M ± SD	9.29 ± 7.53	9.31± 7.71	9.26 ± 7.13	7.82 ± 6.56	11.62 ± 8.25	8.00 ± 0.00	13.00 ± 12.05	8.71 ± 7.73	8.75 ± 7.53	13.07 ± 8.65	9.69 ± 7.43	8.80 ± 5.76	8.00 ± 0.00
	Range	2–24	2–24	2–24	2–24	2–24	8–8	2–24	2–24	2–24	2–24	2–24	2–18	8–8
	ANOVA *p* value	N/A	0.974		0.034				0.483					

### 3.4 Academic Impacts

**FIGURE 3:**
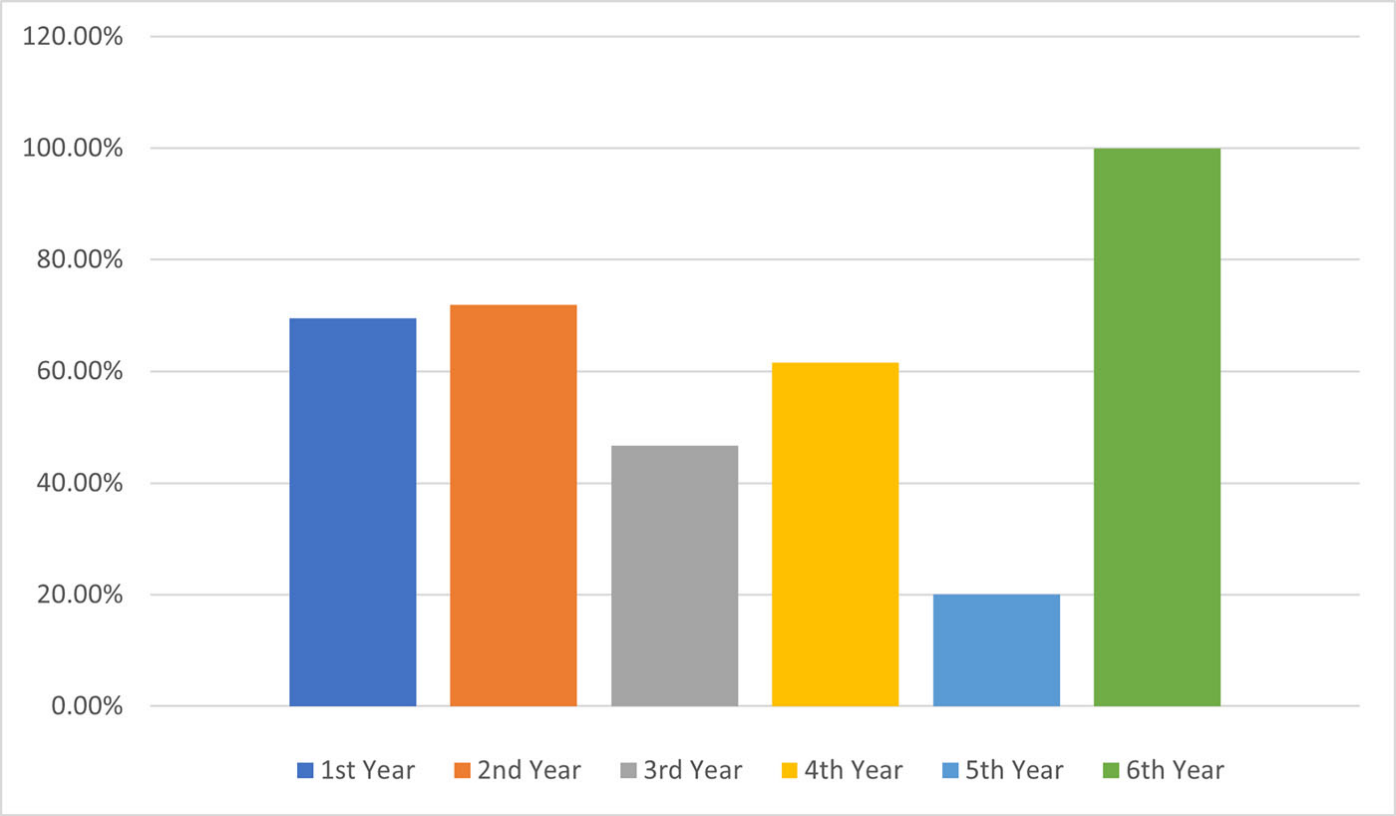
Prevalence of exams triggering migraines across academic years.

**FIGURE 4:**
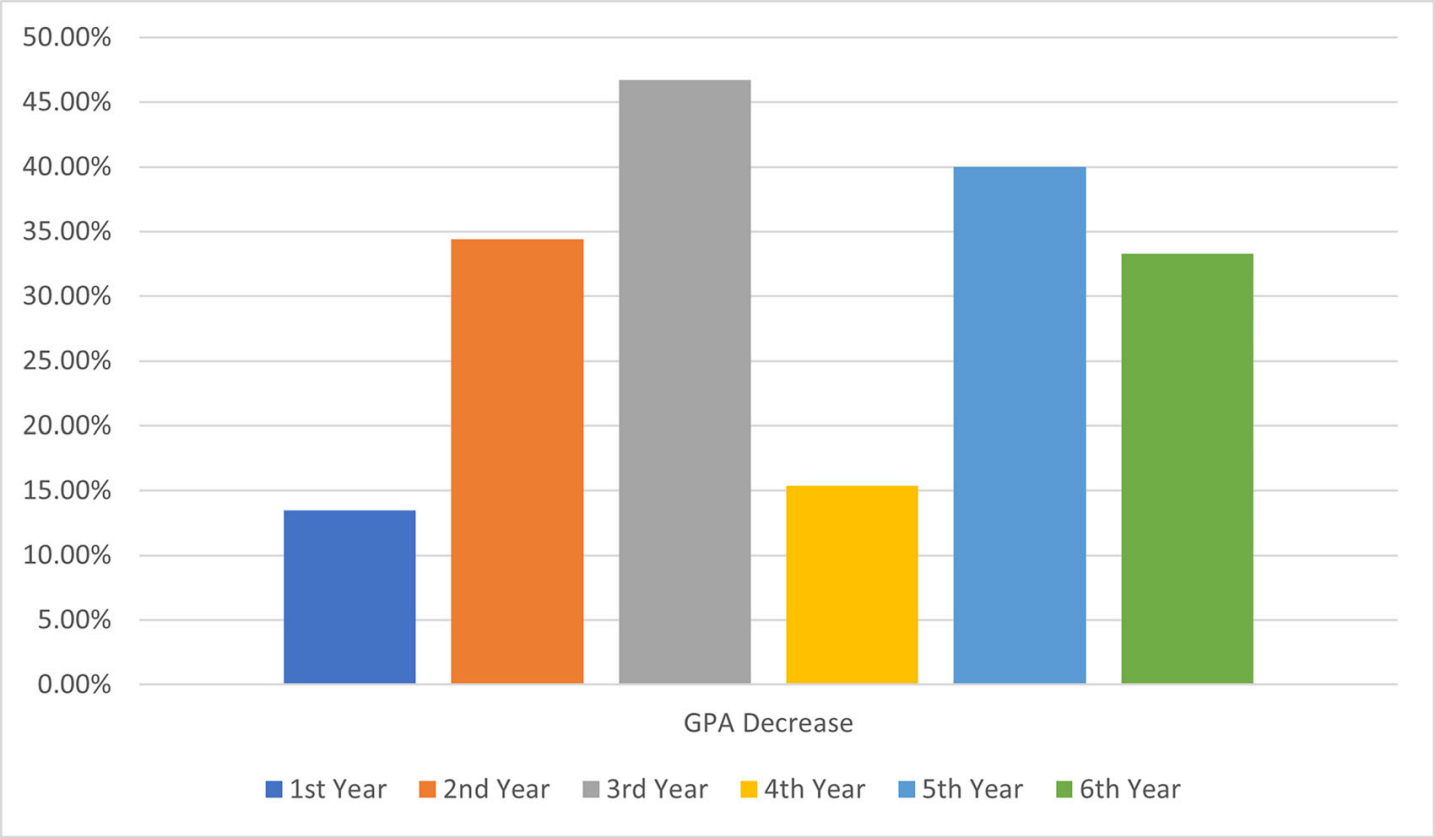
Impact of migraines on GPA decrease by academic year.

**FIGURE 5:**
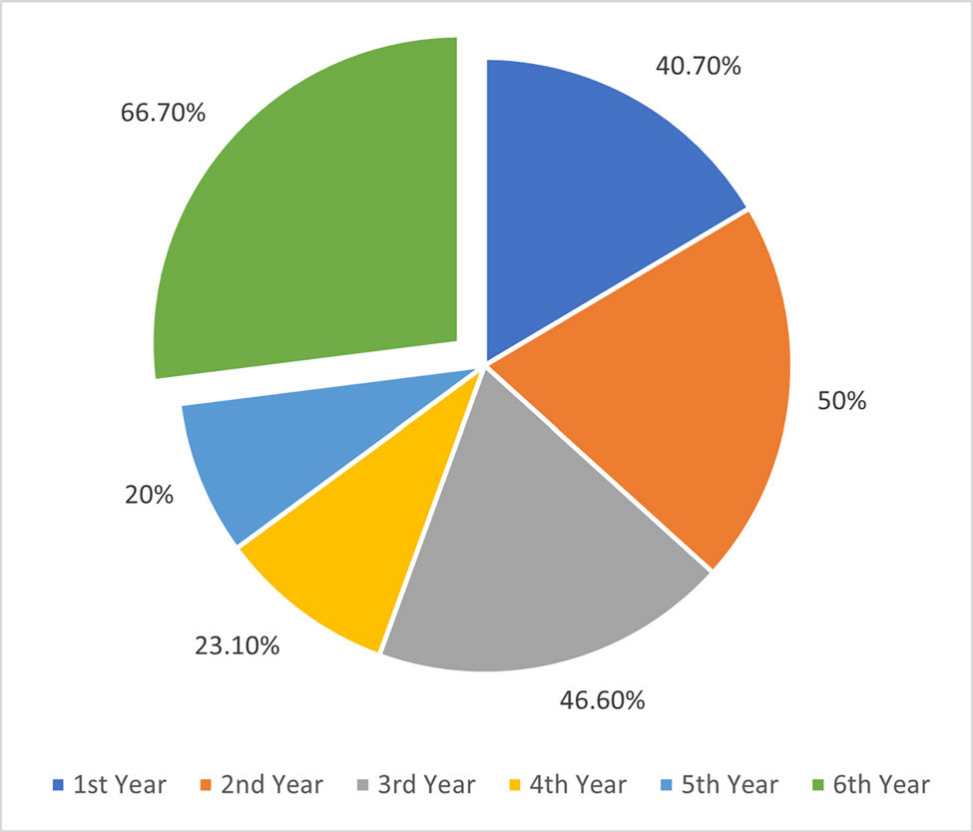
Changes in migraine severity and frequency during exams by academic year.

Among the 130 respondents with a lifetime migraine diagnosis, the impacts of migraine headaches on academic performance and exams were analyzed. Exams were reported to trigger migraine headaches for 86/130 (66%) students. Chi‐square analysis showed migraine triggered by exams had a significant association with the academic year (*χ* ^2^ = 11.256, *p* = 0.047). An increase in migraine headache frequency during exam periods compared to other times was reported by 82/130 (63%) respondents, which was not significantly associated with demographics. Increased migraine intensity/severity during exams was reported by 77/130 (59%) and showed a significant association with the academic year (*χ* ^2^ = 12.564, *p* = 0.028). The average duration of migraine headaches during exams was 4–12 h for 37/130 (29%), <4 h for 36/130 (28%), 12–24 h for 35/130 (27%), and 24+ h for 22/130 (17%). Duration was not significantly associated with gender, age, or academic year. Changes in migraine frequency or severity during exams compared to previous years were highly associated with age (*χ* ^2^ = 37.762, *p* = 0.000019) and academic year (*χ* ^2^ = 85.193, *p* < 0.00001). Regarding academic disruption due to migraine, a productivity limit of ≥1 day was reported by 112/130 (86%), with 2–4 days being the most common (51/130; 39%). The number of exams missed due to migraine differed significantly by academic year (*χ* ^2^ = 47.792, *p* = 0.00045), with more exams missed in later years. Decreases in GPA showed highly significant associations with gender (*χ* ^2^ = 11.120, *p* = 0.0111), age (*χ* ^2^ = 42.803, *p* = 0.00000234), and academic year (*χ* ^2^ = 98.647, *p* < 0.00001). Figure 3 shows the prevalence of exams triggering migraines differed significantly across academic years (*p* = 0.047). Figure 4 illustrates the impact of migraines on GPA decrease, which varied by academic year. Figure 5 displays changes in migraine severity and frequency during exams across academic years. Further details are presented in Appendices A2 and A3.

### 3.5 Coping Strategies

**FIGURE 6:**
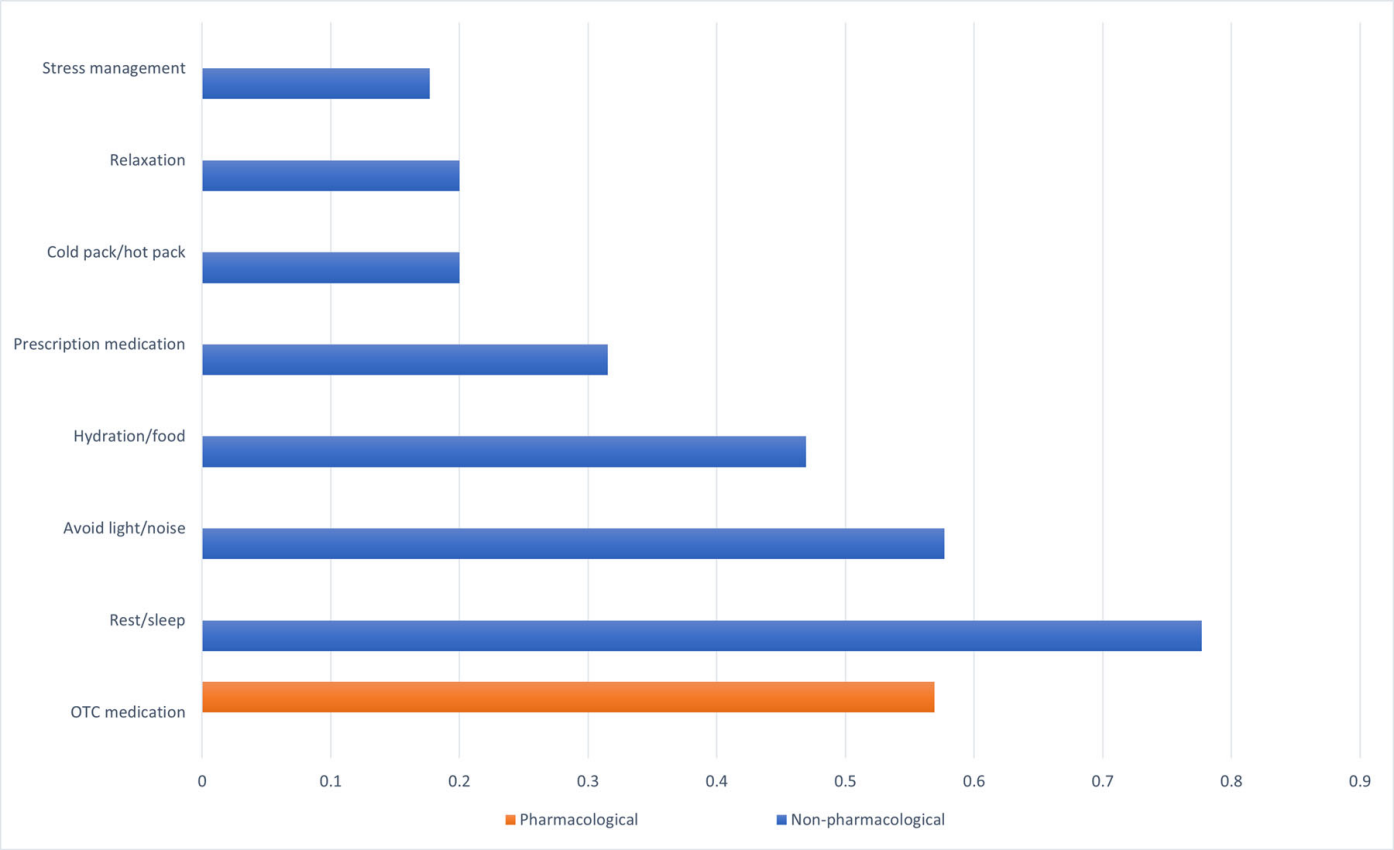
Prevalence of coping strategies among migraine‐diagnosed medical students. OTC, over‐the‐counter.

Among 130 medical students with migraine, the most commonly used coping strategies were rest/sleep (77.7%), avoiding light/noise (57.7%), and over‐the‐counter (OTC) medication (56.9%). Figure 6 shows the prevalence of coping strategies among migraine‐diagnosed medical students. ANOVA found no significant differences in the use of rest/sleep across academic years (*p* = 0.376). However, OTC medication use increased significantly with the academic year (*p* = 0.0028), from 42.4% in the first year to 100% in the sixth year. No significant differences were seen in avoidance of light/noise by academic year (*p* = 0.435).

## 4 Discussion

This cross‐sectional study investigated the prevalence, characteristics, and academic impacts of migraine among medical students at Alfaisal University. The study found a high migraine prevalence, with 36.8% meeting ICHD‐3 criteria, 38.8% identified by ID Migraine screening, and 18.5% fulfilling both criteria (definite migraine). These rates exceed the global migraine prevalence of approximately 11% (GBD 2016 Headache Collaborators 2018) and are higher than those reported in previous studies of medical students in Saudi Arabia (26.3%) (Ibrahim et al. 2017) and Kuwait (27.9%) (Al‐Hashel et al. 2014). The elevated prevalence aligns with research indicating that university students, particularly medical students, are at increased risk for migraine (Al‐Hashel et al. 2014; Ferri‐de‐Barros, Alencar, and Berchielli 2011; Menon and Kinnera 2013; Noor, Sajjad, and Asma 2016; Woldeamanuel and Cowan 2017), likely due to the demanding academic environment and high stress levels (Al‐Hashel et al. 2014). Migraine frequency, severity, and duration were quantified to profile symptoms. The average monthly frequency of 3.5 days, moderate pain severity of 6.4/10, and long mean duration of 9.3 h suggest that students experience recurrent, moderately painful headaches lasting most of the day, which likely impair productivity and quality of life. These findings are consistent with previous studies reporting similar frequency, severity, and duration of migraines among medical students (Amayo, Jowi, and Njeru 2002; Balaban et al. 2012; Ibrahim et al. 2017). The study also found that exams triggered migraines for 66% of students and exacerbated symptoms, including increased headache frequency and severity. This finding is consistent with previous studies reporting that academic stressors and events like examinations can trigger migraine attacks in medical students (Al‐Hashel et al. 2014; Osman Ali, Abbasher Hussien Mohamed Ahmed, and Omer 2022). Exam accommodations, stress management therapy, and strategic medication use around testing periods could help prevent migraine attacks and minimize decreased academic performance. Migraines caused significant academic disruption, limiting productivity for 86% of students. Missed exams and GPA declines increased with advancing academic years, suggesting a growing migraine burden over time. These findings align with prior studies reporting decreased productivity, absenteeism, and psychosocial factors associated with migraines in medical students (Ibrahim et al. 2017; Lebedeva et al. 2017; Menon and Kinnera 2013). Support services to help students manage migraine‐related educational disruption appear warranted. Migraines significantly disrupted academic performance, limiting productivity for 86% of students and increasing missed exams and GPA declines over time. These findings align with prior studies (Ibrahim et al. 2017; Lebedeva et al. 2017; Menon and Kinnera 2013) and highlight the need for support services to manage migraine‐related educational disruption. Our study's findings are consistent with Romozzi et al. (2024), who found high migraine prevalence (26.9%) among Italian medical students and a significant impact on academic performance, with 59.8% experiencing worsening headaches after starting university. These similarities underscore the substantial burden of migraine among medical students across different countries and the need for targeted interventions. The association between migraine and sleep disturbances in medical students is particularly relevant, as this population frequently experiences disrupted circadian rhythms. Recent research suggests common pathophysiological mechanisms (Vgontzas and Pavlović 2018; Waliszewska‐Prosół et al. 2021) and a bidirectional relationship between migraine and sleep disorders (Bertisch et al. 2020). Further research into these mechanisms may provide valuable insights for managing migraine in students with disturbed sleep patterns and could lead to targeted interventions that simultaneously address both conditions, ultimately improving the quality of life and academic performance of affected students. Episodic migraine has been associated with reduced quality of life, increased functional impairment, and higher levels of depression and anxiety symptoms among university students (Romozzi et al. 2024). Students with migraine missed more school days, had more days of impaired functioning at home, and had more medical visits compared to those without migraine (Smitherman, McDermott, and Buchanan 2011), highlighting the significant negative impact of migraine on multiple domains in this understudied population. The most common coping strategies among students with migraine were rest (77.7%), avoidance of light/noise triggers (57.7%), and OTC medication (56.9%). Reliance on OTC medications significantly increased over advancing academic years, consistent with a study of Italian medical students (Romozzi et al. 2024). However, the American Headache Society consensus statement recommends limiting acute treatments to avoid medication overuse headache (Ailani, Burch, and Robbins 2021). Preventive treatments are underutilized in this population (Nicholson et al. 2011). These findings suggest that students may benefit from education on judicious medication use and learning non‐pharmacologic coping skills to promote more effective self‐management and reduce reliance on acute medication alone. Global disparities in headache treatment accessibility, as highlighted by Raffaelli et al. (2023) and Tana et al. (2024), may influence the findings of our study. Medical students in different regions may have varying access to migraine education, preventive strategies, and acute therapies, which could impact their migraine management. The high prevalence of migraine, significant academic impact, and reliance on OTC medications among medical students in our study underscore the need for further research to explore the influence of these global disparities on migraine management in this population. Addressing these disparities requires targeted interventions, such as improving access to migraine education, preventive strategies, and acute therapies tailored to the needs of medical students. Future research should investigate the effectiveness of supportive services and educational programs in improving migraine management and academic outcomes among medical students in different regions, considering the global disparities in headache treatment accessibility. By addressing these disparities and providing targeted interventions, we can work toward improving the quality of life and academic success of medical students with migraines worldwide.

### 4.1 Limitations and Recommendations

This study has several limitations that should be considered when interpreting the results. First, the cross‐sectional design precludes causal inferences about the relationship between migraine and academic performance. Second, the convenience sampling method and the study's focus on a single university may limit the generalizability of the findings to other medical student populations. Third, the reliance on self‐reported data may introduce recall bias and underestimate the true prevalence and impact of migraine. Despite these limitations, our study provides valuable insights into the prevalence, characteristics, and academic impacts of migraine among medical students. We recommend that future research efforts should employ longitudinal designs and probability sampling methods to better understand the causal relationships between migraine and academic performance and to improve the generalizability of the findings. Additionally, we recommend the development and evaluation of targeted interventions, such as migraine education programs, stress management workshops, and preventive treatment strategies, to mitigate the impact of migraine on medical students’ academic performance and overall well‐being. Finally, we recommend that medical schools and universities provide support services and accommodations for students with migraines to help them manage their symptoms and minimize the negative impact on their academic success.

## 5 Conclusion

In conclusion, this study found a high migraine prevalence of 36.8% among medical students, exceeding rates in the general population. Migraines frequently disrupted academic performance and were exacerbated by stressors like exams. These findings indicate a need for multifaceted supportive services and education tailored to the issues faced by students with migraines. Implementation of appropriate accommodations, coping strategies, and self‐care approaches will be instrumental in alleviating migraine burden in this high‐risk cohort. Further research should investigate these interventions longitudinally and across institutions.

## Author Contributions

**Anikó Szabó**: conceptualization, data curation, formal analysis, investigation, methodology, project administration, resources, software, supervision, validation, visualization, writing–original draft, writing–review and editing. **Golam Mahamud**: conceptualization, data curation, formal analysis, investigation, methodology, project administration, resources, software, supervision, validation, visualization, writing–original draft, writing–review and editing. **Faridul Ahsan**: conceptualization, data curation, formal analysis, investigation, methodology, project administration, resources, software, supervision, validation, visualization, writing–original draft, writing–review and editing.

## Ethics Statement

This study was conducted by the Declaration of Helsinki. The study procedures and informed consent process were approved by the Institutional Review Board (IRB) at Alfaisal University before initiation of the research (IRB approval number: 20201).

## Consent

All participants provided informed consent electronically before completing the online questionnaire. The survey was voluntary and anonymous.

## Conflicts of Interest

The authors declare no conflicts of interest.

### Peer Review

The peer review history for this article is available at https://publons.com/publon/10.1002/brb3.70072.

## Data Availability

The data that supports the findings of this study are available in the supplementary material of this article.

## Appendix A

**TABLE A1:** Prevalence and associations of migraine symptoms in the study population.

Symptoms		Overall	Biological sex		Age group				Academic year						
			Male	Female	18–20	21–23	24–26	27+	1st	2nd	3rd		4th	5th	6th
Throbbing, pulsating pain	*N* (%)	100/130 (76.9)	25/35 (71.4)	75/95 (78.9)	60/78 (76.9)	33/42 (78.6)	4/4 (100.0)	3/6 (50.0)	44/59 (74.6)	23/32 (71.9)	12/15 (80.0)	13/13 (100.00		4/5 (80.0)	3/6 (50.0)
	*χ* ^2^	N/A	0.446		3.71				5.00						
	*p* value	N/A	0.5042		0.2940				0.4153						
Pain on one side of the head	*N* (%)	99/130 (76.1)	24/35 (68.6)	75/95 (78.9)	57/78 (73.1)	32/42 (76.2)	4/4 (100.0)	6/6 (100.0)	41/59 (69.5)	28/32 (87.5)	12/15 (80.0)	9/13 (69.2)		5/5 (100.0)	4/6 (66.7)
	*χ* ^2^	N/A	0.998		3.54				6.04						
	*p* value	N/A	0.3176		0.3159				0.3025						
Moderate‐to‐severe intensity	*N* (%)	92/130 (70.8)	29/35 (82.9)	63/95 (66.3)	59/78 (75.6)	28/42 (66.7)	2/4 (50.0)	3/6 (50.0)	46/59 (78.0)	16/32 (50.0)	8/15 (53.3)	13/13 (100.0)		5/5 (100.0)	4/6 (66.7)
	*χ* ^2^	N/A	2.63		3.32				17.84						
	*p* value	N/A	0.1048		0.3446				0.0032						
Increased sensitivity‐to‐light and sound	*N* (%)	87/130 (66.9)	24/35 (68.6)	63/95 (66.3)	51/78 (65.4)	30/42 (71.4)	4/4 (100.0)	2/6 (33.3)	39/59 (66.1)	23/32 (71.9)	7/15 (46.7)	8/13 (61.5)		5/5 (100.0)	5/6 (83.3)
	*χ* ^2^	N/A	0.001		5.50				6.52						
	*p* value	N/A	0.9742		0.1384				0.2585						
Limited your ability to work/study for at least 1 day	*N* (%)	87/130 (66.9)	24/35 (68.6)	63/95 (66.3)	54/78 (69.2)	25/42 (59.5)	4/4 (100.0)	4/6 (66.7)	41/59 (69.5)	21/32 (65.6)	11/15 (73.3)	8/13 (61.5)		3/5 (60.0)	5/6 (83.3)
	*χ* ^2^	N/A	0.0010		3.20				3.85						
	*p* value	N/A	0.9742		0.3613				0.5712						
Nausea and/or vomiting	*N* (%)	50/130 (38.7)	13/35 (37.1)	37/95 (38.9)	26/78 (33.3)	20/42 (47.6)	1/4 (25.0)	3/6 (50.0)	20/59 (33.9)	10/32 (31.2)	6/15 (40.0)	8/13 (61.5)		3/5 (60.0)	3/6 (50.0)
	*χ* ^2^	N/A	0.0		2.998				5.48						
	*p* value	N/A	1.0		0.3919				0.3602						
Worsened by routine activity like walking	*N* (%)	64/130 (49.2)	15/35 (42.9)	49/95 (51.6)	37/78 (47.4)	19/42 (45.2)	2/4 (50.0)	6/6 (100.0)	32/59 (54.2)	14/32 (43.7)	7/15 (46.7)	6/13 (46.1)		3/5 (60.0)	2/6 (33.3)
	*χ* ^2^	N/A	0.469		6.56				1.90						
	*p* value	N/A	0.4936		0.0874				0.8623						

**TABLE A2:** Diverse impacts of migraines on medical students across academic years.

Variables		Overall	Biological sex		Age group				Academic year					
			Male	Female	18–20	21–23	24–26	27+	1st	2nd	3rd	4th	5th	6th
Exam as trigger	Yes	86/130 (66.2%)	21/35 (60.0%)	65/95 (68.4%)	55/78 (70.5%)	24/42 (57.1%)	4/4 (100.0%)	3/6 (50.0%)	41/59 (69.5%)	23/32 (71.9%)	7/15 (46.7%)	8/13 (61.5%)	1/5 (20.0%)	6/6 (100.0%)
	No	44/130 (33.8%)	14/35 (40.0%)	30/95 (31.6%)	23/78 (29.5%)	18/42 (42.86%)	0/4 (0.0%)	3/6 (50.0%)	18/59 (30.5%)	9/32 (28.1%)	8/15 (53.3%)	5/13 (38.5%)	4/5 (80.0%)	0/6 (0.0%)
	*p* value	N/A	0.490		0.177				0.047					
	*χ* ^2^	N/A	0.478		4.931				11.256					
Increase in migraine headache severity	Yes	77/130 (59.2%)	18/35 (51.4%)	59/95 (62.1%)	47/78 (60.3%)	23/42 (54.8%)	4/4 (100.0%)	3/6 (50.0%)	40/59 (67.8%)	16/32 (50.0%)	9/15 (60.0%)	5/13 (38.5%)	1/5 (20.0%)	6/6 (100.0%)
	No	53/130 (40.8%)	17/35 (48.6%)	36/95 (37.9%)	31/78 (39.7%)	19/42 (45.2%)	0/4 (0.0%)	3/6 (50.0%)	19/59 (32.2%)	16/32 (50.0%)	6/15 (40.0%)	8/13 (61.5%)	4/5 (80.0%)	0/6 (0.0%)
	*p* value	N/A	0.369		0.341				0.028					
	*χ* ^2^	N/A	0.806		3.346				12.564					
Increase in migraine headache duration (h)	<4 h	36/130 (27.7%)	9/35 (25.7%)	27/95 (28.4%)	25/78 (32.1%)	10/42 (23.8%)	0/4 (0.0%)	1/6 (16.7%)	21/59 (35.6%)	8/32 (25.0%)	2/15 (13.3%)	4/13 (30.8%)	1/5 (20.0%)	0/6 (0.0%)
	4–12 h	37/130 (28.5%)	14/35 (40.0%)	23/95 (24.2%)	26/78 (33.3%)	14/42 (33.4%)	1/4 (25.0%)	1/6 (16.7%)	14/59 (23.7%)	12/32 (37.6%)	4/15 (26.7%)	3/13 (23.1%)	2/5 (40.0%)	2/6 (33.3%)
	12–24 h	35/130 (26.9%)	7/35 (20.0%)	28/95 (29.5%)	17/78 (21.8%)	9/42 (21.4%)	3/4 (75.0%)	1/6 (16.6%)	14/59 (23.7%)	6/32 (18.7%)	5/15 (33.3%)	5/13 (38.4%)	1/5 (20.0%)	4/6 (66.7%)
	24+ h	22/130 (16.9%)	5/35 14.3%)	17/95 (17.9%)	10/78 (12.8%)	9/42 (21.4%)	0/4 (0.0%)	3/6 (50.0%)	10/59 (17.0%)	6/32 (18.7%)	4/15 (26.7%)	1/13 (7.7%)	1/5 (20.0%)	0/6 (0.0%)
	*p* value	N/A	0.340		0.099				0.506					
	*χ* ^2^	N/A	3.358		14.732				14.261					
Increase in migraine headache frequency (days/month)	Yes	82/130 (63.1%)	20/35 (57.1%)	62/95 (65.3%)	51/78 (65.4%)	24/42 (57.1%)	4/4 (100.0%)	3/6 (50.0%)	40/59 (67.8%)	19/32 (59.4%)	7/15 (46.7%)	9/13 (69.2%)	1/5 (20.0%)	6/6 (100.0%)
	No	48/130 (36.9%)	15/35 (42.9%)	33/95 (34.7%)	27/78 (34.6%)	18/42 (42.9%)	0/4 (0.0%)	3/6 (50.0%)	19/59 (32.2%)	13/32 (40.6%)	8/15 (53.3%)	4/13 (30.8%)	4/5 (80.0%)	0/6 (0.0%)
	*p* value	N/A	0.518		0.309				0.070					
	*χ* ^2^	N/A	0.417		3.595				10.194					
Increase in migraine headache compared to previous years	Yes, more	55/130 (42.3%)	14/35 (40.0%)	41/95 (43.2%)	35/78 (44.9%)	15/42 (35.7%)	2/4 (50.0%)	3/6 (50.0%)	24/59 (40.7%)	16/32 (50.0%)	7/15 (46.6%)	3/13 (23.1%)	1/5 (20.0%)	4/6 (66.7%)
	Yes, less	8/130 (6.2%)	2/35 (5.7%)	6/95 (6.3%)	1/78 (1.3%)	5/42 (11.9%)	2/4 (50.0%)	0/6 (0.0%)	0/59 (0.0%)	1/32 (3.1%)	1/15 (6.7%)	4/13 (30.8%)	0/5 (0.0%)	2/6 (33.3%)
	No change	35/130 (26.9%)	10/38 (28.6%)	25/95 (26.3%)	14/78 (17.9%)	18/42 (42.9%)	0/4 (0.0%)	3/6 (50.0%)	4/59 (6.8%)	15/32 (46.9%)	6/15 (40.0%)	6/13 (46.1%)	4/5 (80.0%)	0/6 (0.0%)
	N/A‐first year	32/130 (24.6%)	9/35 (25.7%)	23/95 (24.2%)	28/78 (35.9%)	4/42 (9.5%)	0/4 (0.0%)	0/6 (0.0%)	31/59 (52.5%)	0/32 (0.0%)	1/15 (6.7%)	0/13 (0.0%)	0/5 (0.0%)	0/6 (0.0%)
	*p* value	N/A	0.986		0.000019				*p* < 0.00001					
	*χ* ^2^	N/A	0.147		37.762				85.193					

**TABLE A3:** Statistical breakdown of migraine impact on academic performance across demographics.

Variables		Overall	Biological sex		Age group				Academic year					
			Male	Female	18–20	21–23	24–26	27+	1st	2nd	3rd	4th	5th	6th
Exams missed	0	118/130 (90.8%)	32/35 (91.4%)	86/95 (90.5%)	71/78 (91.0%)	37/42 (88.1%)	4/4 (100.0%)	6/6 (100.0%)	55/59 (93.2%)	29/32 (90.6%)	12/15 (80.0%)	13/13 (100.0%)	5/5 (100.0%)	4/6 (66.7%)
	1	5/130 (3.9%)	1/35 (2.9%)	4/95 (4.2%)	4/78 (5.1%)	1/42 (2.4%)	0/4 (0.0%)	0/6 (0.0%)	1/59 (1.7%)	1/32 (3.1%)	3/15 (20.0%)	0/13 (0.0%)	0/5 (0.0%)	0/6 (0.0%)
	2	2/130 (1.5%)	0/35 (0.0%)	2.95 (2.1%)	1/78 (1.3%)	1/42 (2.4%)	0/4 (0.0%)	0/6 (0.0%)	0/59 (0.0%)	2/32 (6.3%)	0/15 (0.0%)	0/13 (0.0%)	0/5 (0.0%)	0/6 (0.0%)
	3	2/130 (1.5%)	2/35 (5.7%)	0/95 (0.0%)	2/78 (2.6%)	0/42 (0.0%)	0/4 (0.0%)	0/6 (0.0%)	2/59 (3.4%)	0/32 (0.0%)	0/15 (0.0%)	0/13 (0.0%)	0/5 (0.0%)	0/6 (0.0%)
	4+	3/130 (2.3%)	0/35 (0.0%)	3/95 (3.2%)	0/78 (0.0%)	3/42 (7.1%)	0/4 (0.0%)	0/6 (0.0%)	1/59 (1.7%)	0/32 (0.0%)	0/15 (0.0%)	0/13 (0.0%)	0/5 (0.0%)	2/6 (33.3%)
	*p* value	N/A	0.116		0.696				0.00045					
	*χ* ^2^	N/A	7.395		9.080				47.792					
Productivity limit (day)	0	18/130 (13.9%)	6/35 (17.1%)	12/95 (12.6%)	11/78 (14.1%)	7/42 (16.7%)	0/4 (0.0%)	0/6 (0.0%)	6/59 (10.2%)	5/32 (15.6%)	4/15 (26.7%)	2/13 (15.4%)	1/5 (20.0%)	0/6 (0.0%)
	1	35/130 (26.9)	9/35 (25.7%)	26/95 (27.4%)	23/78 (29.5%)	10/42 (23.8%)	2/4 (50.0%)	0/6 (0.0%)	19/59 (32.2%)	3/32 (9.4%)	8/15 (53.3%)	2/13 (15.4%)	1/5 (20.0%)	2/6 (33.4%)
	2–4	51/130 (39.2%)	14/35 (40.0%)	37/95 (39.0%)	29/78 (37.2%)	14/42 (33.3%)	2/4 (50.0%)	6/6 (100.0%)	23/59 (39.0%)	17/32 (53.1%)	2/15 (13.3%)	6/13 (46.1%)	1/5 (20.0%)	2/6 (33.3%)
	5+	26/130 (20.0%)	6/35 (17.2%)	20/95 (21.0%)	15/78 (19.2%)	11/42 (26.2%)	0/4 (0.0%)	0/6 (0.0%)	11/59 (18.6%)	7/32 (21.9%)	1/15 (6.7%)	3/13 (23.1%)	2/5 (40.0%)	2/6 (33.3%)
	*p* value	N/A	0.895		0.144				0.162					
	*χ* ^2^	N/A	0.605		13.428				20.262					
GPA decrease	Yes	32/130 (24.6%)	11/35 (31.4%)	21/95 (22.1%)	11/78 (14.1%)	18/42 (42.9%)	0/4 (0.0%)	3/6 (50.0%)	8/59 (13.5%)	11/32 (34.4%)	7/15 (46.7%)	2/13 (15.4%)	2/5 (40.0%)	2/6 (33.3%)
	No	26/130 (20.0%)	3/35 (8.6%)	23/95 (24.2%)	15/78 (19.2%)	9/42 (21.4%)	2/4 (50.0%)	3/6 (50.0%)	4/59 (6.8%)	9/32 (28.1%)	5/15 (33.3%)	5/13 (38.5%)	1/5 (20.0%)	2/6 (33.3%)
	Variable	25/130 (19.3%)	12/35 (34.3%)	13/95 (13.7%)	19/78 (11.5%)	1/42 (26.2%)	2/4 (50.0%)	0/6 (0.0%)	0/59 (0.0%)	12/32 (37.5%)	3/15 (20.0%)	6/13 (46.1%)	2/5 (40.0%)	2/6 (33.4%)
	N/A‐first year	47/130 (36.1%)	9/35 (25.7%)	38/95 (40.0%)	43/78 (55.1%)	4/42 (9.5%)	0/4 (0.0%)	0/6 (0.0%)	47/59 (79.7%)	0/32 (0.0%)	0/15 (0.0%%)	0/13 (0.0%)	0/5 (0.0%)	0/5 (0.0%)
	*p* value	N/A	0.0111		0.00000234				*p* < 0.00001					
	*χ* ^2^	N/A	11.120		42.803				98.647					

## References

[brb370072-bib-0001] Ailani, J. , R. C. Burch , and M. S. Robbins . 2021. “Board of Directors of the American Headache Society. The American Headache Society Consensus Statement: Update on Integrating New Migraine Treatments Into Clinical Practice.” Headache 61, no. 7: 1021–1039. 10.1111/head.14153. 34160823

[brb370072-bib-0002] Al‐Hashel, J. Y. , S. F. Ahmed , R. Alroughani , and P. J. Goadsby . 2014. “Migraine Among Medical Students in Kuwait University.” Journal of Headache and Pain 15, no. 1: 26. 10.1186/1129-2377-15-26. 24886258 PMC4029817

[brb370072-bib-0003] Amayo, E. O. , J. O. Jowi , and E. K. Njeru . 2002. “Headache Associated Disability in Medical Students at the Kenyatta National Hospital, Nairobi.” East African Medical Journal 79, no. 10: 519–523. 10.4314/eamj.v79i10.8813. 12635756

[brb370072-bib-0004] Balaban, H. , M. Semiz , I. A. Şentürk , et al. 2012. “Migraine Prevalence, Alexithymia, and Post‐Traumatic Stress Disorder Among Medical Students in Turkey.” Journal of Headache and Pain 13, no. 6: 459–467. 10.1007/s10194-012-0452-7. 22535148 PMC3464464

[brb370072-bib-0005] Bertisch, S. M. , W. Li , C. Buettner , et al. 2020. “Nightly Sleep Duration, Fragmentation, and Quality and Daily Risk of Migraine.” Neurology 94, no. 5: e489–e496. 10.1212/WNL.0000000000008740. 31843807 PMC7080287

[brb370072-bib-0006] Bigal, M. E. , J. M. Bigal , M. Betti , C. A. Bordini , and J. G. Speciali . 2001. “Evaluation of the Impact of Migraine and Episodic Tension‐Type Headache on the Quality of Life and Performance of a University Student Population.” Headache 41, no. 7: 710–719. 10.1046/j.1526-4610.2001.041007710.x. 11554960

[brb370072-bib-0007] Ferri‐de‐Barros, J. E. , M. J. Alencar , L. F. Berchielli , and L. C. Castelhano Junior . 2011. “Headache Among Medical and Psychology Students.” Arquivos De Neuro‐Psiquiatria 69, no. 3: 502–508. 10.1590/s0004-282x2011000400018. 21755130

[brb370072-bib-0008] GBD 2016 Headache Collaborators . 2018. “Global, Regional, and National Burden of Migraine and Tension‐Type Headache, 1990–2016: A Systematic Analysis for the Global Burden of Disease Study 2016.” [published correction appears in Lancet Neurol. 2021 Dec;20(12)e7. doi: 10.1016S1474‐4422(21)00380‐X] Lancet Neurology 17, no. 11: 954–976. 10.1016/S1474-4422(18)30322-3. 30353868 PMC6191530

[brb370072-bib-0009] Headache Classification Committee of the International Headache Society (IHS) . 2018. “The International Classification of Headache Disorders, 3rd Edition.” Cephalalgia 38, no. 1: 1–211. 10.1177/0333102417738202. 29368949

[brb370072-bib-0010] Ibrahim, N. K. , A. K. Alotaibi , A. M. Alhazmi , R. Z. Alshehri , R. N. Saimaldaher , and M. A. Murad . 2017. “Prevalence, Predictors and Triggers of Migraine Headache Among Medical Students and Interns in King Abdulaziz University, Jeddah, Saudi Arabia.” Pakistan Journal of Medical Sciences 33, no. 2: 270–275. 10.12669/pjms.332.12139. 28523020 PMC5432687

[brb370072-bib-0011] Lebedeva, E. R. , N. R. Kobzeva , D. V. Gilev , N. V. Kislyak , and J. Olesen . 2017. “Psychosocial Factors Associated With Migraine and Tension‐Type Headache in Medical Students.” Cephalalgia 37, no. 13: 1264–1271. 10.1177/0333102416678389. 27919020

[brb370072-bib-0012] Lipton, R. B. , D. Dodick , R. Sadovsky , et al. 2003. “A Self‐Administered Screener for Migraine in Primary Care: The ID Migraine Validation Study.” Neurology 61, no. 3: 375–382. 10.1212/01.wnl.0000078940.53438.83. 12913201

[brb370072-bib-0013] Menon, B. , and N. Kinnera . 2013. “Prevalence and Characteristics of Migraine in Medical Students and Its Impact on Their Daily Activities.” Annals of Indian Academy of Neurology 16, no. 2: 221–225. 10.4103/0972-2327.112472. 23956569 PMC3724079

[brb370072-bib-0014] Nicholson, R. A. , D. C. Buse , F. Andrasik , and R. B. Lipton . 2011. “Nonpharmacologic Treatments for Migraine and Tension‐Type Headache: How to Choose and When to Use.” Current Treatment Options in Neurology 13, no. 1: 28–40. 10.1007/s11940-010-0102-9. 21080124

[brb370072-bib-0015] Noor, T. , A. Sajjad , and A. Asma . 2016. “Frequency, Character and Predisposing Factor of Headache Among Students of Medical College of Karachi.” JPMA the Journal of the Pakistan Medical Association 66, no. 2: 159–164. 26819160

[brb370072-bib-0016] Osman Ali, M. M. , K. Abbasher Hussien Mohamed Ahmed , and M. E. A. Omer . 2022. “Prevalence of Migraine Headaches and Their Impact on the Academic Performance of Sudanese Medical Students Using ID‐Migraine Test as a Screening Tool: A Cross‐Sectional Study.” Brain and Behavior 12, no. 5: e2588. 10.1002/brb3.2588. 35451242 PMC9120720

[brb370072-bib-0017] Raffaelli, B. , E. Rubio‐Beltrán , S. J. Cho , et al. 2023. “Health Equity, Care Access and Quality in Headache—Part 2.” Journal of Headache and Pain 24, no. 1: 167. 10.1186/s10194-023-01699-7. 38087219 PMC10717448

[brb370072-bib-0018] Romozzi, M. , V. Trigila , G. Cuffaro , P. Calabresi , and C. Vollono . 2024. “Primary Headaches Prevalence, Characteristics, and Healthcare Utilization in Italian Medical Students.” Neurological Sciences 45, no. 6: 2893–2897. 10.1007/s10072-024-07375-1. 38342838

[brb370072-bib-0019] Smitherman, T. A. , R. Burch , H. Sheikh , and E. Loder . 2013. “The Prevalence, Impact, and Treatment of Migraine and Severe Headaches in the United States: A Review of Statistics From National Surveillance Studies.” Headache 53, no. 3: 427–436. 10.1111/head.12074. 23470015

[brb370072-bib-0020] Smitherman, T. A. , M. J. McDermott , and E. M. Buchanan . 2011. “Negative Impact of Episodic Migraine on a University Population: Quality of Life, Functional Impairment, and Comorbid Psychiatric Symptoms.” Headache 51, no. 4: 581–589. 10.1111/j.1526-4610.2011.01857.x. 21457242

[brb370072-bib-0021] Souza‐e‐Silva, H. R. , and P. A. Rocha‐Filho . 2011. “Headaches and Academic Performance in University Students: A Cross‐Sectional Study.” Headache 51, no. 10: 1493–1502. 10.1111/j.1526-4610.2011.02012.x. 22082420

[brb370072-bib-0022] Tana, C. , B. Raffaelli , M. N. P. Souza , et al. 2024. “Health Equity, Care Access and Quality in Headache—Part 1.” Journal of Headache and Pain 25, no. 1: 12. 10.1186/s10194-024-01712-7. 38281917 PMC10823691

[brb370072-bib-0023] Tfelt‐Hansen, P. , J. Pascual , N. Ramadan , et al. 2012. “Guidelines for Controlled Trials of Drugs in Migraine: Third Edition. A Guide for Investigators.” Cephalalgia 32, no. 1: 6–38. 10.1177/0333102411417901. 22384463

[brb370072-bib-0024] Vgontzas, A. , and J. M. Pavlović . 2018. “Sleep Disorders and Migraine: Review of Literature and Potential Pathophysiology Mechanisms.” Headache 58, no. 7: 1030–1039. 10.1111/head.13358. 30091160 PMC6527324

[brb370072-bib-0025] Waldie, K. E. , M. Hausmann , B. J. Milne , and R. Poulton . 2002. “Migraine and Cognitive Function: A Life‐Course Study.” Neurology 59, no. 6: 904–908. 10.1212/wnl.59.6.904. 12297575

[brb370072-bib-0026] Waliszewska‐Prosół, M. , M. Nowakowska‐Kotas , J. Chojdak‐Łukasiewicz , and S. Budrewicz . 2021. “Migraine and Sleep—An Unexplained Association?.” International Journal of Molecular Sciences 22, no. 11: 5539. 10.3390/ijms22115539. 34073933 PMC8197397

[brb370072-bib-0027] Woldeamanuel, Y. W. , and R. P. Cowan . 2017. “Migraine Affects 1 in 10 People Worldwide Featuring Recent Rise: A Systematic Review and Meta‐Analysis of Community‐Based Studies Involving 6 Million Participants.” Journal of the Neurological Sciences 372: 307–315. 10.1016/j.jns.2016.11.071. 28017235

